# Cinnamaldehyde as a Natural Antimicrobial in Donkey Semen: Efficacy and Sperm Cytotoxicity

**DOI:** 10.1111/rda.70250

**Published:** 2026-09-30

**Authors:** Carlos Montero‐Palma, Antonio Romero‐Salmoral, Pilar Vallejo‐Soto, Blenda Rodrigues Nunes Vilela, Verónica Pérez, Francisco Crespo‐Castejón, Isabel Ortiz, Manuel Hidalgo, Jesús Dorado, Inmaculada Luque, Lidia Gómez‐Gascón

**Affiliations:** ^1^ Grupo de Reproducción Veterinaria. Dpto. Medicina y Cirugía Animal. Universidad de Córdoba Córdoba Spain; ^2^ Dpto. Sanidad Animal. Unidad de Investigación Competitiva (UIC) Zoonosis y Enfermedades Emergentes ENZOEM, Campus de Excelencia Internacional Agroalimentaria ‘CeiA3’, Universidad de Córdoba Córdoba Spain; ^3^ Centro Militar de Cría Caballar de Ávila, Cría Caballar de Las Fuerzas Armadas Ávila Spain

**Keywords:** antimicrobial susceptibility, bacteria, Cinnamaldehyde, donkey, MIC, sperm, toxicity

## Abstract

Bacterial contamination of donkey semen samples is a major concern due to its deleterious effects on sperm quality; this issue is exacerbated during semen storage. Therefore, evaluating the effect of antimicrobials on sperm is of utmost importance. This study identified microorganisms present in donkey semen and evaluated the antimicrobial activity of cinnamaldehyde, determined by the Minimum Inhibitory Concentration (MIC), and its effects on sperm quality. Fourteen semen samples from seven donkeys were analysed, identifying 31 bacterial isolates representing 21 different species. Cinnamaldehyde showed antimicrobial activity (MIC_50_ = 0.06 mg/mL; MIC_90_ = 4 mg/mL). Reactive oxygen species levels in sperm were decreased, suggesting an antioxidant effect. However, high concentrations of cinnamaldehyde reduced sperm motility. In conclusion, cinnamaldehyde is a promising antimicrobial for semen preservation; however, further studies are needed to ensure antimicrobial efficacy and sperm functionality.

## 1 Introduction

Different microorganisms from the skin and the animal's environment can colonize the mucosa of the distal reproductive tract and be transferred to the semen during ejaculation. Additionally, semen collection and processing increase the risk of bacterial contamination. To mitigate this, antibiotics are commonly incorporated into extenders during semen preparation of samples for artificial insemination. However, antibiotic exposure may negatively impact sperm quality parameters (Zabala et al. 2025).

Consequently, alternatives to conventional antibiotics that do not compromise sperm quality have been investigated. Cinnamaldehyde, the main component of cinnamon essential oil, has attracted attention for its antibacterial and antioxidant properties, which may help preserve sperm by mitigating damage caused by reactive oxygen species (ROS) (Heidarzadeh and Tabatabaei Vakili 2025).

Although horses and donkeys share similar reproductive characteristics, differences in disease susceptibility and reproductive management exist (Carleton et al. 2015). Research on the donkey semen microbiome remains limited. Therefore, this study aimed to (i) identify the main microorganisms present in donkey semen and (ii) evaluate the in vitro antimicrobial activity of cinnamaldehyde against these isolates as well as its potential cytotoxic effects on sperm as a preliminary step toward its possible use in semen extenders as a safe alternative to conventional antibiotics.

## 2 Materials and Methods

### 2.1 Microbiological Analysis

A total of 14 semen samples were collected from 7 donkeys between 2022 and 2025 under aseptic conditions to minimize the risk of external contamination. Samples were diluted 1:10 in sterile buffered peptone water to reduce seminal plasma interference and enable accurate bacterial growth and colony quantification (Zabala et al. 2024). Subsequently, they were plated on MacConkey, Mannitol, Blood, Columbia with 5% of sterile defibrinated sheep blood, and Sabouraud agar (Oxoid Ltd). Plates were incubated at 37°C for 72 h under aerobic conditions, and Sabouraud agar at 30°C for 1 week (Corona and Cherchi 2009). Presumptive identification of isolates was performed by Gram staining, oxidase and catalase tests, and glucose fermentation, and was confirmed by MALDI‐TOF analysis (Bruker MALDI Biotyper) with score values ≥ 2.0 (Pérez‐Sancho et al. 2017).

Diluents, culture media, and materials were sterilized by autoclaving or exposure to ultraviolet light, and all laboratory procedures were conducted in laminar flow cabinets. Sterility testing of the culture media and PBS was performed before use (Zabala et al. 2024).

### 2.2 Antimicrobial Susceptibility Test

Antimicrobial susceptibility was determined by the broth microdilution method following Clinical and Laboratory Standards Institute (CLSI 2016). Cinnamaldehyde (≥ 95% purity, Sigma‐Aldrich, St. Louis, MO, USA) was serially twofold diluted to final concentrations of 64–0.06 mg/mL in U‐bottom 96‐well microtiter plates (Lab‐Center, Spain) and mixed with an equal volume of bacterial inoculum (5 × 10^5^ CFU/mL) in Mueller–Hinton broth (MHB). Plates were incubated at 37°C for 24 h under aerobic conditions with positive and negative controls included. The minimum inhibitory concentration (MIC), as well as MIC_50_ and MIC_90_ values (concentrations inhibiting 50% and 90% of isolates), were determined.

### 2.3 Cinnamaldehyde Dilutions and Semen Processing

Cinnamaldehyde was diluted in semen extender (INRA96; IMV Technologies, L'Aigle, France) obtaining three working solutions equilibrated at 17°C (Vallejo‐Soto et al. 2025). Six semen samples were collected from three donkeys available throughout the entire study period. Two sampling sessions were conducted on separate dates to assess the repeatability of the results. Samples were centrifuged, resuspended to 50 × 10^6^ sperm/mL and stored at 17°C for 24 h. Subsequently, samples were diluted with cinnamaldehyde solutions to final concentrations of 0, 0.06 (MIC_50_), 0.24 (MIC_50_ × 4) and 4 (MIC_90_) mg/mL. Sperm parameters were evaluated immediately after exposure (t0) and after 24 h (t24) at 17°C. Assessed parameters included sperm motility by computer‐assisted sperm analysis (SCA v.6.5; Microptic, Barcelona, Spain), morphology by formalin fixation (Hernández‐Avilés et al. 2023), plasma and acrosomal membrane integrity (PI/FITC‐PNA) (Ortiz et al. 2021), and relative ROS (rROS) levels in viable spermatozoa (DCFH‐DA) (Medica et al. 2025) by flow cytometry (Accuri C6 Plus; BD, Franklin Lakes, NJ, USA).

### 2.4 Statistical Analysis

The effect of cinnamaldehyde concentrations on sperm parameters was analysed using a general linear model with Tukey's post hoc test (SAS Institute Inc., Cary, NC, USA). Statistical significance was set at *p* < 0.05.

## 3 Results

### 3.1 Microbiological Analysis

Bacterial growth was detected in all samples, while no fungal isolates were recovered. A total of 31 isolates were obtained, including 20 Gram‐positive strains: *Bacillus* (*n* = 4), *Streptococcus* (*n* = 3), *Staphylococcus* (*n* = 4), *Aerococcus* (*n* = 1), *Corynebacterium* (*n* = 2), *Enterococcus* (*n* = 4) and *Globicatella* (*n* = 2), and 11 Gram‐negative strains: *Enterobacter* (*n* = 2), *Stenotrophomonas* (*n* = 2), *Pseudomonas* (*n* = 2), *Escherichia* (*n* = 3), *Klebsiella* (*n* = 1) and *Providencia* spp. (*n* = 1). These isolates belonged to 13 different genera (Figure 1).

**FIGURE 1:**
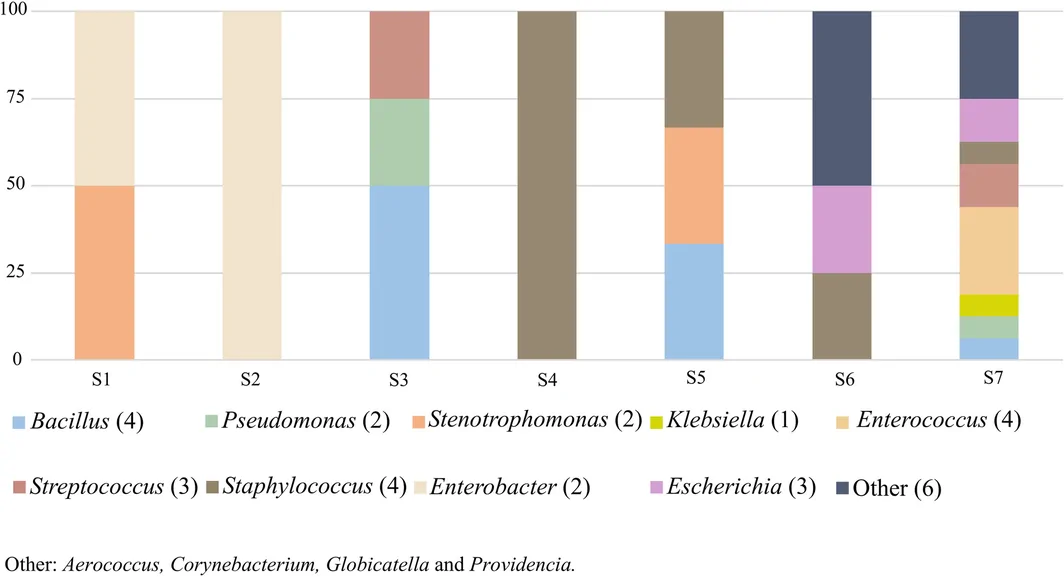
Relative abundance (%) of bacterial genera isolated from seven donkey semen samples (S1‐7). The number of isolates per genus is shown in parentheses.

### 3.2 Cinnamaldehyde Antimicrobial Test

The antimicrobial activity of cinnamaldehyde against all isolates showed MIC_50_ and MIC_90_ values of 0.06 mg/mL and 4 mg/mL, respectively, indicating broad‐spectrum activity with variability between Gram‐positive and Gram‐negative bacteria (Figure 2).

**FIGURE 2:**
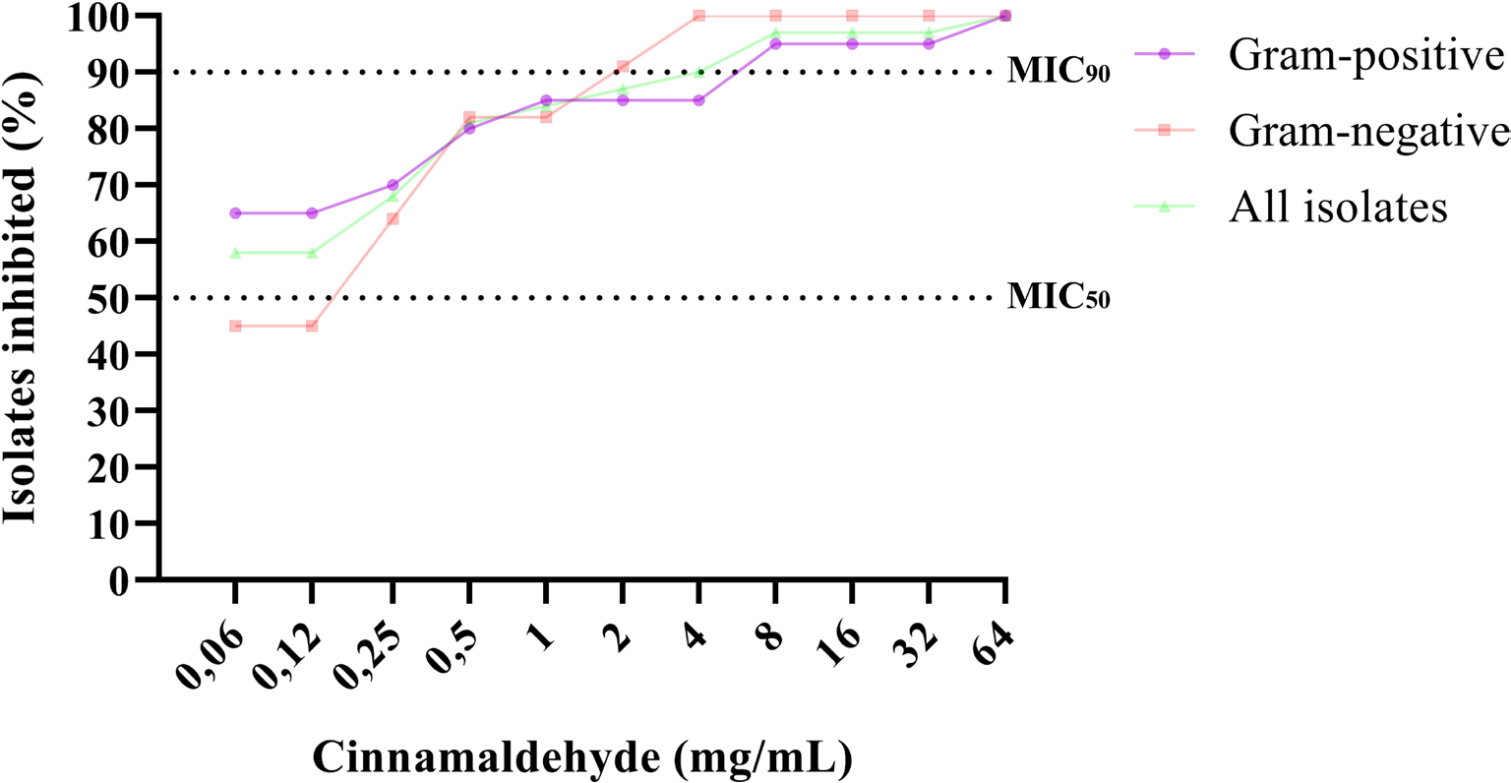
Cumulative distribution of minimum inhibitory concentrations (MICs) of cinnamaldehyde against Gram‐positive (*n* = 20), Gram‐negative (*n* = 11), and all bacterial isolates from donkey semen.

### 3.3 Sperm Cytotoxicity Tests

Sperm motility, viability, and rROS levels at t0 and t24 after exposure to different concentrations of cinnamaldehyde are summarized in Figure 3.

**FIGURE 3:**
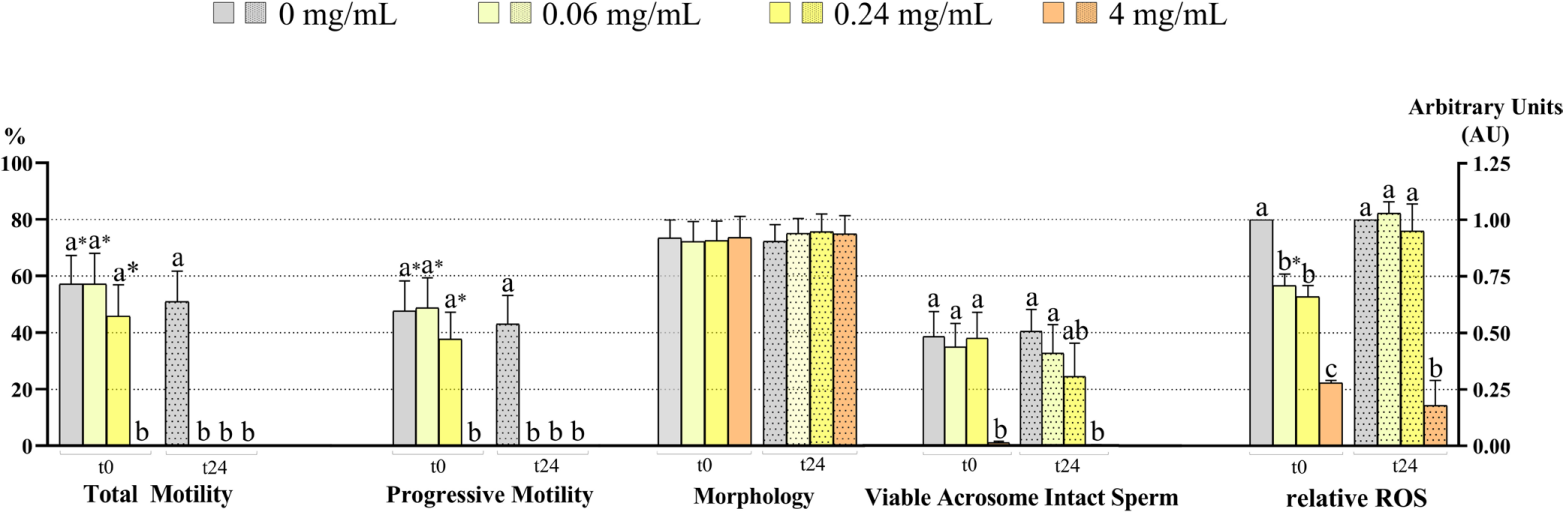
Effect of different cinnamaldehyde concentrations (0–4 mg/mL) on sperm motility, viability, and rROS levels at 0 (t0) and 24 h (t24). Different superscripts (a‐c) indicate significant differences (*p* < 0.05) among cinnamaldehyde concentrations within time point. Asterisks indicate significant differences (*p* < 0.05) between time points for the same cinnamaldehyde concentration.

## 4 Discussion

Collected semen can contain microorganisms from various sources. In this study, bacteria from genital microbiota (*Streptococcus, Enterococcus, Staphylococcus, Enterobacter, Escherichia*) and environmental contaminants (*Corynebacterium, Bacillus, Aerococcus, Globicatella, Klebsiella, Pseudomonas, Stenotrophomonas, Providencia*) have been identified in donkey semen, indicating high microbial diversity (Corona and Cherchi 2009). Although the pathogenic relevance of these microorganisms may vary among host species, some detected genera, such as Klebsiella and Pseudomonas, include species associated with reproductive tract infections, endometritis, abortion, and reduced fertility in equids. Bacterial contamination of semen has also been associated with decreased sperm quality, as certain bacteria can impair motility, compromise membrane integrity, and increase DNA fragmentation through toxins and reactive oxygen species (ROS) (Malaluang et al. 2021; Youssef et al. 2026). However, the relationship between seminal microbiota and sperm quality remains unclear and likely depends on bacterial composition, abundance, and interactions within the seminal environment.

Cinnamaldehyde showed antibacterial activity against bacterial isolates in our study, inhibiting most strains at 0.06 mg/mL, in agreement with previous reports (Firmino et al. 2018). Its effect may vary among species, with Gram‐negative bacteria generally exhibiting higher resistance. This is attributed to the presence of phospholipids, lipopolysaccharides, and proteins in their outer membrane, which act as a barrier limiting the entry of lipophilic compounds (Pourkhosravani et al. 2021).

Exposure of donkey sperm to low concentrations of cinnamaldehyde did not affect sperm motility or viability at t0, whereas higher concentrations reduced both parameters in a dose‐dependent manner. These effects were more pronounced after 24 h, as previously reported (Qadeer et al. 2025), suggesting that high cinnamaldehyde concentrations may disrupt plasma membrane lipids, altering membrane fluidity and compromising its integrity and sperm motility. ROS levels decreased at 0.06 and 0.24 mg/mL, suggesting a short‐term antioxidant effect. Cinnamaldehyde may neutralize ROS through its α,β‐unsaturated group and activate cellular defence mechanisms (Heidarzadeh and Tabatabaei Vakili 2025). However, excessive antioxidant activity may impair mitochondrial oxidative phosphorylation and ATP production, leading to reduced flagellar motility and loss of sperm motility after 24 h (Shi et al. 2020).

Although cinnamaldehyde exhibited promising antibacterial activity against a broad range of seminal bacterial isolates (MIC_50_: 0.06 mg/mL), its negative effects on sperm quality indicate that further optimization is required before practical application in semen preservation. Future research should focus on the development of formulations that preserve antimicrobial efficacy while minimizing spermicidal effects, including synergistic combinations with antibiotics, organic acids, essential oils, and other natural antimicrobial compounds to reduce the effective dose. Notably, the spermicidal activity observed at higher concentrations suggests potential applicability as a natural spermicidal agent; however, additional studies are required to confirm its efficacy and safety. In addition, studies involving a larger number of animals are needed to account for biological variability and to further assess the safety and applicability of cinnamaldehyde in donkey semen preservation.

## 5 Conclusions

A diverse range of bacterial genera can be isolated from donkey semen, some of which may impair sperm quality. The present study demonstrated that cinnamaldehyde exhibits antimicrobial activity against these bacteria while preserving sperm motility and viability at low concentrations (0.06 mg/mL) during short exposure periods. Additionally, a reduction in ROS levels was observed, suggesting a potential antioxidant effect. However, prolonged exposure negatively affected motility. Further research is required to determine optimal formulations that ensure antimicrobial efficacy while maintaining sperm functionality.

## Author Contributions

C.M.‐P., A.R.‐S., P.V.‐S., B.R.N.V., and V.P. performed the experiments. C.M.‐P., I.O., I.L., and L.G.‐G. analysed the results and prepared the manuscript. C.M.‐P., F.C.‐C., M.H., J.D., I.O., I.L., and L.G.‐G. contributed to the experimental design, data analysis, preparation, and final approval of the manuscript. M.H. and J.D. are the PIs of the project.

## Funding

This study was supported by the project grant PID2020‐116090RB‐I00 from MICIN (Ministerio de Ciencia e Innovación), which also supported CM‐P. PV‐S was supported by the Spanish FPU fellowship (FPU23/03472) from Ministerio de Universidades. BRNV was funded in part by the Coordenacão de Aperfeicoamento de Pessoal de Nivel Superior‐Brazil (CAPES). Funding for open access charge: Universidad de Córdoba/CBUA.

## Conflicts of Interest

The authors declare no conflicts of interest.

## Data Availability

The data that support the findings of this study are available from the corresponding author upon reasonable request.
